# Impacts of early insulin treatment vs glimepiride in diabetic patients with background metformin therapy: A nationwide retrospective cohort study

**DOI:** 10.1097/MD.0000000000025085

**Published:** 2021-03-05

**Authors:** Fu-Shun Yen, Chih-Cheng Hsu, Yuan-Chih Su, James Cheng-Chung Wei, Chii-Min Hwu

**Affiliations:** a Dr. Yen's Clinic, No. 15, Shanying Road, Gueishan District, Taoyuan; b Institute of Population Health Sciences, National Health Research Institutes, Zhunan; c Department of Health Services Administration, China Medical University, Taichung; d Department of Family Medicine, Min-Sheng General Hospital, Taoyuan; e Management Office for Health Data, China Medical University Hospital; f College of Medicine, China Medical University, Taichung; g Institute of Medicine, Chung Shan Medical University; h Department of Medicine, Chung Shan Medical University Hospital; i Graduate Institute of Integrated Medicine, China Medical University, Taichung; j Department of Medicine, National Yang-Ming University School of Medicine; k Section of Endocrinology and Metabolism, Department of Medicine, Taipei Veterans General Hospital, Taipei, Taiwan.

**Keywords:** propensity score, mortality, cause of death

## Abstract

Type 2 diabetes mellitus (T2DM) is a progressive disease. After metformin failure, the addition of insulin or sulfonylureas might increase the risk of hypoglycemia and cardiovascular (CV) morbidity. Here, the risk of all-cause mortality was compared between early insulin treatment and glimepiride use in T2DM patients with background metformin therapy.

We conducted a 9-year retrospective cohort study from the population-based National Health Insurance Research Database in Taiwan. A total of 2054 patients with T2DM under insulin or glimepiride treatment were enrolled during 2004 to 2012. Overall event rates of all-cause mortality were compared between 1027 insulin users and 1027 matched glimepiride users.

After the propensity score matching, the mortality rates were 72.5 and 4.42 per 1000 person-years for insulin users and glimepiride users. The adjusted hazard ratio of mortality was 14.47 (95% CI: 8.64–24.24; *P* value <.001) as insulin compared with glimepiride users. The insulin users had significantly higher risk of CV death (adjusted hazard ratio 7.95, 95% CI 1.65–38.3, *P* = .01) and noncardiovascular death (adjusted hazard ratio 14.9, 95% CI 8.4–26.3, *P* < .001).

The nationwide study demonstrated that metformin plus insulin therapy was associated with higher risk of all-cause mortality.

## 1 Introduction

Type 2 diabetes mellitus had the property of progressive β**-**cell failure. Upon diagnosis of diabetes, most patients were found to have a 50% decrease in their insulin secretion with a relentless 4% decline per year.^[1]^ At last, most patients would require insulin treatment, alone or in combination with oral hypoglycemic agents.^[2]^ United Kingdom Prospective Diabetes Study (UKPDS) suggested that earlier initiation of treatment was associated with better outcome.^[3]^ There were also some reports which demonstrated that early intensive insulin treatment of hyperglycemia had favorable outcomes on recovery and maintenance of β-cell function with lengthened glycemic remission as compared with treatments of oral hypoglycemic agents.^[4–6]^ The epidemiological studies disclosed that Asian diabetic patients had the characteristics of declining β-cell function more sharply than insulin sensitivity with age and rapid oral drug failure^[7]^; therefore the promotion of early insulin treatment in Asian patients was reasonable.

The UKPDS study^[8]^ disclosed that intensive therapy with insulin or sulfonylureas (SU) had similar effect. The ORIGIN trial demonstrated that the use of basal insulin was safe on CV outcomes and cancer occurrence.^[9]^ But Holden et al^[10]^ reported 6484 T2DM patients who progressed to treatment with insulin monotherapy, the adjusted hazard ratio (aHR) in relation to 1-unit increases in insulin dose was 1.54 for all-cause mortality and 1.35 for cancer. A retrospective cohort study of 63,579 diabetic patients treated in general practice disclosed that the aHR of association with serious atherosclerotic vascular disease of the heart was 1.3 for insulin.^[11]^ One report from the Euro Heart Survey on Diabetes and the Heart disclosed that insulin therapy might relate to a more serious prognosis in patients with coronary artery disease (CAD) and diabetes.^[12]^

Sulfonylureas were prescribed in very large quantities due to their low cost and rapid lowering of glucose level. In recent years, there were always debates on the detrimental effect of SU in diabetic patients, including its possible CV morbidity and mortality;^[13,14]^ some studies suggested avoiding the use of SU in diabetes treatment, especially after insulin initiation.^[15]^ Both insulin and SU had the propensities of hypoglycemia, body weight increase and possibly CV injury. Colayco et al^[16]^ conducted a nested case-control study to compare insulin plus oral medications (including SU) vs no diabetes medications, and found that the insulin plus oral medications group had higher risk of getting CV events (odds ratio = 2.56). Currie et al^[17]^ compared insulin based regimens with metformin plus SU, and found that the hazard ratio for all-cause mortality in people given insulin-based regimens vs those given combined oral agents was 1.49. Though, there were some reports implying the benefits of early insulin therapy, but these were all short-term clinical studies without long-term outcomes.^[18]^ Many observational studies have also indicated that insulin therapy is more risky than oral hypoglycemic agents.^[10–12,16,17]^ And there are currently few risk comparisons of using insulin vs sulfonylurea after metformin use. Therefore, we conducted this cohort study to see the risks of all-cause mortality between insulin and glimepiride use in T2DM patients with background metformin therapy.

## 2 Materials and methods

### 2.1 Data source

NHIRD contained the medical data of National Health Insurance (NHI), which had been implemented since March 1995, and over 99% of Taiwan residents had joined the NHI.^[19]^ We used the data from Longitudinal Health Insurance database 2000 (LHID2000), a sub-dataset of NHIRD. The LHID2000 recorded the medical care data of 1 million people. The demographics of the LHID2000 were similar to the whole Taiwan population. In the LHID2000, the medical information included encrypted identification, demographics, the International Classification of Diseases, 9th Revision, Clinical Modification (ICD-9-CM) codes, surgery records and drug records.

### 2.2 Study design

Using the National Health Insurance Research Database (NHIRD), we investigated the difference of diabetes treatments in this population-based cohort study. We compared the adults of aged 18 to 100 years with metformin plus insulin vs metformin plus glimepiride therapy. Our study was approved by the Research Ethics Committee of China Medical University and Hospital, Taichung, Taiwan (CMUH104-REC2–115 (CR-2)). Our research was granted a waiver of informed consent. The information and records of patients were de-identified prior to analysis and encrypted the identification of each participant.

### 2.3 Study population

The study population consisted of diabetic patients (ICD-9-CM: 250.x) with metformin treatment excluding type 1 diabetic patients (250.1x). We categorized diabetic patients as insulin cohort or glimepiride cohort with underlying metformin treatment in 2004 to 2012 years. The insulin cohort contained type 2 diabetic patients with metfomin plus insulin treatment and exlcuded all concurrent sulfonylureas users. The glimepiride cohort contained type 2 diabetic patients with metfomin plus glimepiride treatmen. The index date was the time of receiving insulin or glimepiride.

### 2.4 Identification of confounders

The demographics of gender and age were confounders of this study. Baseline comorbidity was defined as having following diseases before the index date: coronary artery disease (ICD-9-CM: 410–414), stroke (ICD-9-CM: 430–438), hypertension (ICD-9-CM: 401–405), and dyslipidemia (ICD-9-CM: 272). We used the Charlson Comorbidity Index (CCI) to quantify patients’ comorbidity profiles.^[20]^ We defined the severity of diabetes according to Diabetes Complications Severity Index (DCSI) score.^[21]^ The CCI and DCSI scores were calculated using participant status 1 year before the index date. We also considered other drugs for diabetes such as thiazolidinediones (TZDs), alpha-glucosidase inhibitors (AGIs), and dipeptidyl peptidase-4 inhibitors (DPP-4i) as possible confounders.

### 2.5 The primary outcome and causes of death

The primary outcome of this study was all- cause mortality. The observation period started from the index date to the withdrawal from the NHI or 31st December, 2013 or the date of death, whichever came first. We assessed the last primary diagnosis of discharge 3 months before death, to search for the causes of death.^[22]^ The causes of CV death were according to the Standardized Definitions for End Point Events in Cardiovascular Trials.^[23]^ Death due to other causes was defined as noncardiovascular death. The cases that we could not get last primary diagnosis 3 months before death were defined as undetermined.

### 2.6 Statistical methods

To match the insulin cohort and glimepiride cohort, we performed 1:1 propensity score matching^[24]^ with the criteria of age, gender, comorbidities, CCI scores, DCSI scores and other antidiabetic drugs use. We summarized the variables in insulin and glimepiride cohorts, and compared those between 2 groups using Chi-Squared test for dichotomous variables and two-sample Student *t* test for continuous variables. In this study, we used simple and multivariable Cox proportional hazards regression models to estimate the excess risk of all-cause mortality for the insulin cohort compared to the glimepiride cohort. We calculated the crude and adjusted HR with corresponding 95% confidence intervals (95% CIs). The multivariable Cox proportional hazards regression models were done under the control of age, gender, comorbidities, other antidiabetic drugs, CCI and DCSI scores. The stratified analysis of each variable using Cox proportional hazards regression model was conducted in the outcome of mortality. The cumulative incidences of survival rate of each cohort were estimated using Kaplan–Meier method and examined by log-rank test.

All statistical analyses were conducted by the statistical software package, SAS, version 9.4 (SAS Institute, Inc., Cary, NC). *P* value less than .05 was the threshold of statistical significance in our study.

## 3 Results

**Figure 1:**
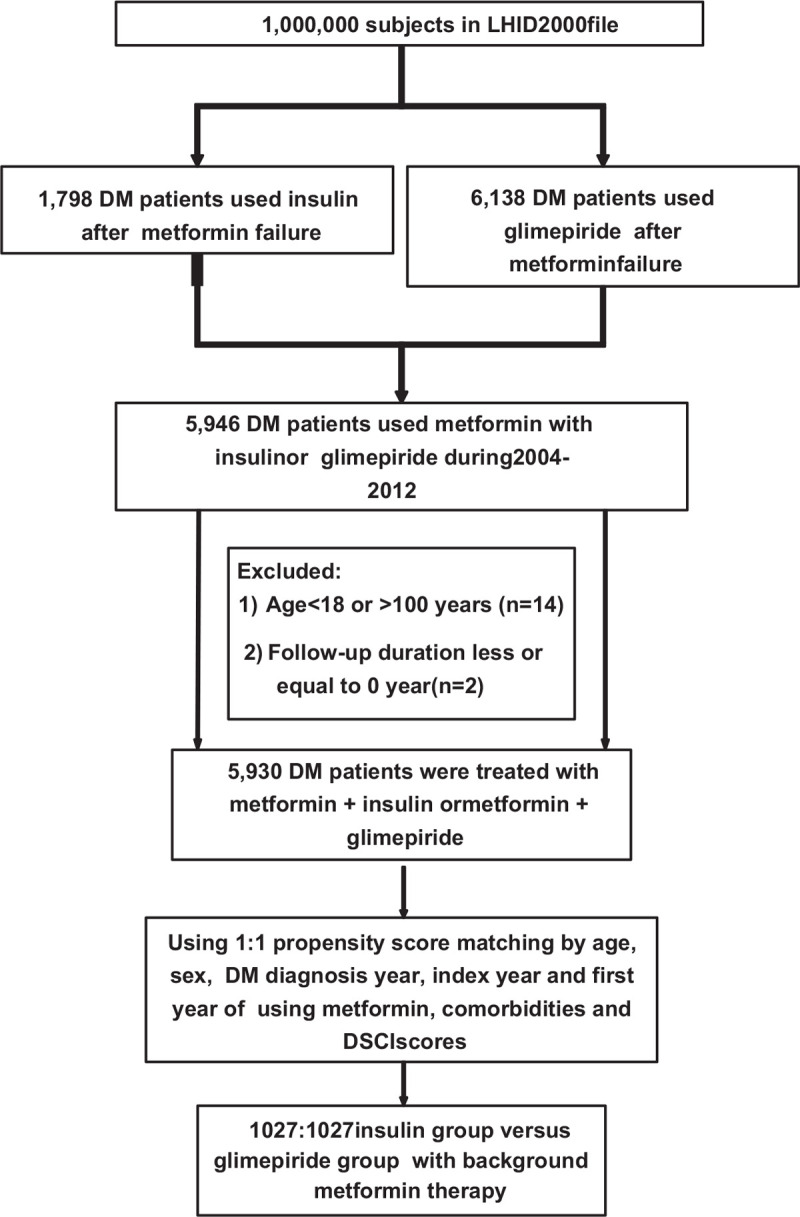
The flow chart that identified the number of patients and study design.

From the data file of 2000 to 2012 years of LHID2000 (Fig. 1), there were 1798 patients injected insulin without using sulfonyureas and 6138 were treated with glimepiride with background metformin therapy. After excluding and matching, we enrolled 1027 diabetic patients in insulin and glimepiride cohorts, respectively. The mean (median) follow-up duration was 4.47 (3.16) and 4.72 (3.06) years for metformin plus insulin group and metformin plus glimepiride group. Men comprised 54.2% in the insulin group, and 55% in the glimepiride group (Table 1). Patients over 65 years old had the highest proportion in insulin cohort (49.4%), but a greater proportion was found in patients between 41 and 65 years old in the glimepiride cohort (48.7%). Between the 2 groups, only the distribution of age classification was notably different (*P* value = .011), there was no significant difference in mean age between the 2 populations (*P* value = .29).

**Table 1:** Demographic characteristics and co-morbidity of insulin group and glimepiride group in diabetic patients with background metformin therapy.

	Glimepiride (N = 1027)	Insulin (N = 1027)	
	n (%)	n (%)	*P* value
Gender			.72^∗^
Women	462 (45)	470 (45.8)	
Men	565 (55)	557 (54.2)	
Age, years			.011^∗^
≤40	37 (3.6)	63 (6.1)	
41–65	500 (48.7)	457 (44.5)	
>65	490 (47.7)	507 (49.4)	
Mean (SD)	63.5 (12.5)	64.2 (14.6)	.29^†^
Baseline comorbidity			
Coronary artery disease	431 (42)	423 (41.2)	.72^∗^
Stroke	337 (32.8)	311 (30.3)	.22^∗^
Hypertension	746 (72.6)	752 (73.2)	.77^∗^
Dyslipidemia	654 (63.7)	649 (63.2)	.82^∗^
CCI scores			.24^∗^
0, 1	346 (33.7)	381 (37.1)	
2, 3	407 (39.6)	378 (36.8)	
>3	274 (26.7)	268 (26.1)	
DSCI scores			.59^∗^
0	703 (68.5)	683 (66.5)	
1	229 (22.3)	238 (23.2)	
≥2	95 (9.3)	106 (10.3)	
DM drugs			
TZDs	117 (11.4)	107 (10.4)	.48^∗^
Alpha glucosidase inhibitors	260 (25.3)	239 (23.3)	.28^∗^
DPP-4i	208 (20.3)	197 (19.2)	.54^∗^
Mean DM duration, days (medium)	1724 (1106)	1630 (1152)	

The mortality risk was higher by 14.19-fold in the insulin cohort, as compared with the glimepiride cohort (95% CI = 8.48–23.75, *P* value < .001; Table 2). The gender of male also had higher risk of mortality (men aHR = 1.47, 95% CI = 1.09–1.99, *P* value = .01). Diabetic patients using alpha-glucosidase inhibitors or DPP-4i had lower risk of mortality (alpha-glucosidase inhibitors aHR = 0.54, 95% CI = 0.34–0.84, *P* value = .01; DPP-4i aHR = 0.26, 95% CI = 0.13–0.52, *P* value <.001).

**Table 2:** Cox model measured hazard ratio and 95% confidence intervals of death associated treatment groups and covariates in diabetic patients with background metformin therapy.

	Event no.	Crude			Adjusted		
Characteristics	(n = 182)	HR	(95% CI)	*P* value	HR	(95% CI)	*P* value
Treatment							
Glimepiride	16	1	reference		1	reference	
Insulin	166	13.33	(7.97–22.28)	<.001	14.19	(8.48–23.75)	<.001
Gender							
Women	73	1	reference		1	reference	
Men	109	1.27	(0.95–1.71)	.11	1.47	(1.09–1.99)	.01
Age, years							
≤40	7	1	reference		1	reference	
41–65	50	0.74	(0.34–1.64)	.47	0.78	(0.35–1.78)	.56
>65	125	1.97	(0.92–4.22)	.08	1.73	(0.76–3.95)	.19
Baseline comorbidity							
Coronary artery disease							
No	92	1	reference		1	reference	
Yes	90	1.43	(1.07–1.91)	.02	1.1	(0.79–1.54)	.56
Stroke							
No	114	1	reference		1	reference	
Yes	68	1.34	(0.99–1.81)	.06	1.08	(0.79–1.48)	.64
Hypertension							
No	32	1	reference		1	reference	
Yes	150	1.8	(1.23–2.63)	.003	1.33	(0.87–2.03)	.19
Dyslipidemia							
No	76	1	reference		1	reference	
Yes	106	0.79	(0.59–1.06)	.11	0.73	(0.53–0.99)	.05
CCI scores							
0, 1	50	1	reference		1	reference	
2, 3	73	1.38	(0.96–1.98)	.08	1.29	(0.88–1.87)	.19
>3	59	1.66	(1.14–2.42)	.01	1.37	(0.91–2.07)	.14
DSCI scores							
0	116	1	reference		1	reference	
1	46	1.21	(0.86–1.7)	.28	0.92	(0.63–1.34)	.67
≥2	20	1.17	(0.73–1.88)	.52	0.66	(0.39–1.11)	.11
DM drugs							
TZDs							
No	172	1	reference		1	reference	
Yes	10	0.43	(0.23–0.81)	.01	0.75	(0.39–1.43)	.38
AGIs							
No	159	1	reference		1	reference	
Yes	23	0.43	(0.28–0.66)	<.001	0.54	(0.34–0.84)	.01
DPP-4i							
No	173	1	reference		1	reference	
Yes	9	0.19	(0.1–0.37)	<.001	0.26	(0.13–0.52)	<.001

**Figure 2:**
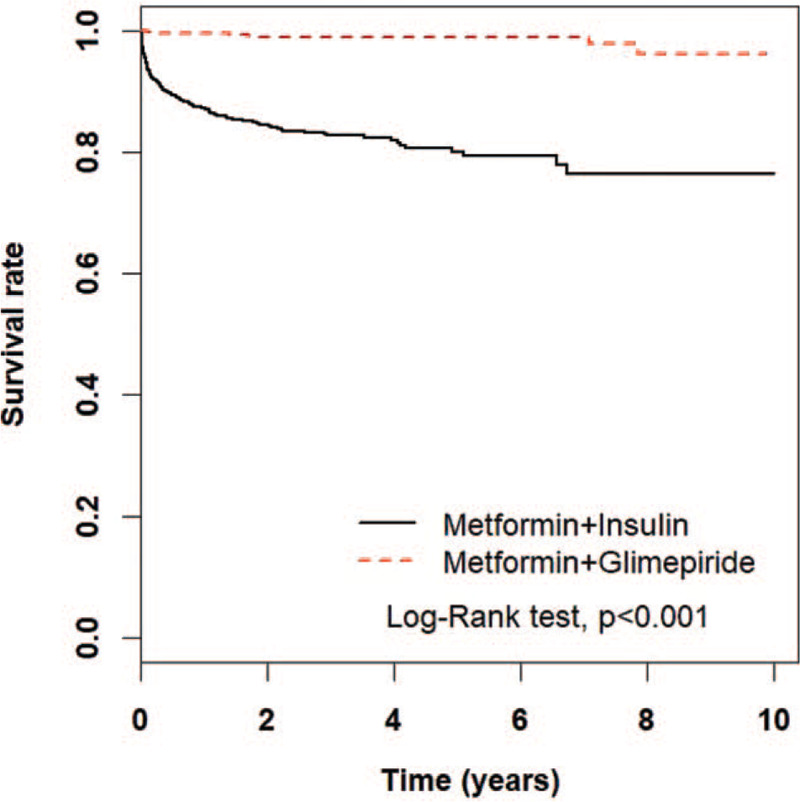
The estimated survival rates between the insulin group and glimepiride group in diabetic patients with background metformin therapy by Kaplan–-Meier method.

The overall mortality rates in insulin and glimepiride cohort were 72.5 and 4.42 per 1000 person-years (Table 3). During the study period, the cumulative survival rate among insulin cohort was significantly lower than the cumulative survival rate among glimepiride cohort (*P* value <.001; Fig. 2). Table 3 showed the subgroup analysis of mortality of metformin plus insulin vs metform plus glimepiride. Notably, metfomin plus insulin had higher risk of mortlity among all the subgroups of genders, age, comorbidites, CCI scores DCSI scores, and other DM drugs use.

**Table 3:** Cox model of measured hazard ratio and 95% confidence intervals of mortality between insulin group and glimepiride group with background metformin therapy and covariates.

	Metformin						Insulin vs glimepiride	
	Glimepiride (n = 1027)			Insulin (n = 1027)				
Variables	Event	Person years	IR^†^	Event	Person years	IR^†^	Crude HR (95% CI)	Adjusted HR (95% CI)
Overall	16	3623	4.42	166	2290	72.5	13.33 (7.97–22.28)^∗∗∗^	14.47 (8.64–24.24)^∗∗∗^
Gender								
Women	3	1664	1.8	70	1097	63.8	29.03 (9.13–92.24)^∗∗∗^	31.36 (9.83–100.05)^∗∗∗^
Men	13	1959	6.64	96	1193	80.4	9.7 (5.43–17.33)^∗∗∗^	10.91 (6.08–19.57)^∗∗∗^
Age, years								
≤40	0	148	0	7	163	43.0	20291126.21 (0-.)	128397943.32 (0-.)
41–65	2	1818	1.1	48	1179	40.7	30.03 (7.3–123.57)^∗∗∗^	30.4 (7.37–125.34)^∗∗∗^
>65	14	1658	8.44	111	949	117	11.18 (6.4–19.52)^∗∗∗^	11.88 (6.78–20.82)^∗∗∗^
Comorbidity								
Coronary artery disease								
No	6	2202	2.72	86	1398	61.5	17.78 (7.77–40.72)^∗∗∗^	18.62 (8.11–42.75)^∗∗∗^
Yes	10	1421	7.04	80	892	89.7	10.68 (5.53–20.63)^∗∗∗^	12.96 (6.65–25.24)^∗∗∗^
Stroke								
No	7	2468	2.84	107	1663	64.4	18.18 (8.46–39.09)^∗∗∗^	19.25 (8.94–41.44)^∗∗∗^
Yes	9	1155	7.79	59	627	94.0	10.16 (5.02–20.55)^∗∗∗^	11.11 (5.43–22.74)^∗∗∗^
Hypertension								
No	1	1011	0.99	31	660	46.9	38.09 (5.2–279.31)^∗∗∗^	37.16 (5.05–273.56)^∗∗∗^
Yes	15	2612	5.74	135	1630	82.8	11.74 (6.88–20.03)^∗∗∗^	12.79 (7.48–21.87)^∗∗∗^
Dyslipidemia								
No	7	1396	5.01	69	794	86.9	13.38 (6.14–29.17)^∗∗∗^	14.54 (6.6–32.02)^∗∗∗^
Yes	9	2227	4.04	97	1497	64.8	13.4 (6.77–26.55)^∗∗∗^	14.77 (7.44–29.35)^∗∗∗^
CCI scores								
0, 1	2	1302	1.54	48	938	51.2	26.68 (6.48–109.87)^∗∗∗^	24.55 (5.93–101.6)^∗∗∗^
2, 3	5	1433	3.49	68	820	82.9	19.17 (7.72–47.6)^∗∗∗^	21.04 (8.45–52.39)^∗∗∗^
>3	9	887	10.1	50	532	93.9	7.66 (3.76–15.6)^∗∗∗^	8.72 (4.22–17.99)^∗∗∗^
DSCI scores								
0	11	2464	4.46	105	1493	70.3	12.7 (6.82–23.67)^∗∗∗^	13.98 (7.47–26.14)^∗∗∗^
1	2	789	2.53	44	510	86.3	26.89 (6.51–111.02)^∗∗∗^	33.66 (8.07–140.48)^∗∗∗^
≥2	3	369	8.12	17	287	59.3	6.28 (1.84–21.45)^∗∗^	6.42 (1.85–22.31)^∗∗^
DM drugs								
TZDs								
No	15	3112	4.82	157	2002	78.4	13.3 (7.82–22.6)^∗∗∗^	14.35 (8.43–24.43)^∗∗∗^
Yes	1	511	1.96	9	288	31.2	13.92 (1.75–110.92)^∗^	44.28 (3.43–572.18)^∗∗^
AGIs								
No	14	2699	5.19	145	1705	85.1	13.23 (7.64–22.92)^∗∗∗^	14.46 (8.33–25.09)^∗∗∗^
Yes	2	924	2.16	21	585	35.9	13.9 (3.25–59.44)^∗∗∗^	14.57 (3.37–63.07)^∗∗∗^
DPP-4i								
No	16	2801	5.71	157	1779	88.3	12.76 (7.63–21.36)^∗∗∗^	13.61 (8.12–22.83)^∗∗∗^
Yes	0	822	0	9	512	17.6		

The major identifiable causes of death of the insulin cohort included: 10 (0.97%) CV death (1 ischemic heart disease, 3 sudden cardiac deaths, 1 heart failure, 2 stroke, 3 CV hemorrhage); 142 (13.832%) noncardiovascular death (52 cancers, 90 others); and 14 (1.36%) undetermined cases. The major identifiable causes of death of the glimepiride cohort included: 2 (0.20%) CV death (1 heart failure, 1 strokes); 13 (1.27%) noncardiovascular death (3 cancers, 10 others); and 1 (0.10%) undetermined cases (Table 4). The insulin users, as compared with the glimepiride users, had significantly higher risk of CV death (adjusted hazard ratio 7.95, 95% CI 1.65–38.3, *P* = .01) and noncardiovascular death (adjusted hazard ratio 14.9, 95% CI 8.4–26.3, *P* < .001, Table 4).

**Table 4:** The causes of death of insulin vs glimepiride groups in patients with background metformin therapy.

	Metformin		Insulin vs glimepiride	
	Glimepiride n (%)	Insulin n (%)	Adjusted HR (95% CI)	*P* value
Causes of CV death	2 (0.20)	10 (0.97)	7.95 (1.65–38.3)	.01
Ischemic heart disease	0	1		
Sudden cardiac death	0	3		
Heart failure	1	1		
Stroke	1	2		
Cardiovascular procedure				
Cardiovascular hemorrhage	0	3		
Other cardiovascular causes				
Non-cardiovascular causes of death	13 (1.27)	142 (13.83)	14.9 (8.4–26.3)	<.001
Cancers	3	52		
others	10	90		
Undetermined	1 (0.10)	14 (1.36)	23.9 (3.1–184)	.002

## 4 Discussion

We used a one to one propensity score matching to compare the risk of all-cause mortality between insulin and glimepiride users with background metformin therapy. Our results disclosed that insulin users had significantly higher risk of all-cause mortality, significantly higher risk of CV and noncardiovascular death. This overwhelming high risk of mortality was apparent across genders, age groups, baseline comorbidities, concurrent antidiabetic drugs use and DCSI scores.

The UKPDS study^[8]^ disclosed that intensive therapy with insulin or SU had similar effect, but this study was not designed to compare these 2 regimens. The ORIGIN trial demonstrated that the use of basal insulin was safe on CV outcomes, but the dose of insulin was very low (0.4 μ/kg/day) in quite early diabetic stage. Eleven percent of the placebo group also used exogenous insulin, which made the comparison not so adequate.^[9]^ Margolis et al^[11]^ conducted a retrospective cohort study and disclosed that insulin (aHR = 1.2) based treatment (including SU) was associated with an increased risk of myocardial infarction, and the risk increased with longer use. Colayco et al^[16]^ conducted a nested case-control study to compare insulin plus oral medications with no diabetic medications, the insulin based group had higher risk (odds ratio = 2.56) of getting CV events. The post-hoc analysis from the Diabetes Mellitus Insulin-Glucose Infusion in Acute Myocardial Infarction (DIGAMI) 2 trial on extended long-term outcome disclosed that insulin based treatment might be associated with increased risk of nonfatal cardiac events.^[25]^ Hall et al^[26]^ reported that adding insulin after 1 oral glucose-lowering drug (OGLD), when compared with adding another OGLD, had poor macrovascular outcomes. Currie et al^[17]^ compared insulin based therapy with metformin plus SU regimens and found that insulin-based treatment had higher risk of all-cause mortality (HR = 1.49). Gamble et al^[27]^ used the administrative databases of Saskatchewan Health to survey the cumulative insulin exposure based on total insulin dispensations per year. They observed a significant and graded association between mortality risk and insulin exposure. These were all insulin combined with oral medications including SU compared with oral medications or no medication, and showed that insulin based managements had higher risk of CV events and all-cause mortality.

As for the early use of insulin after metformin failure without adding SU compared with metformin plus SU. Roumie et al^[28]^ reported the intensification of metformin with insulin vs sulfonylureas was associated with an increased risk of a composite of nonfatal CV outcomes and all-cause mortality in white male veterans. Mogensen et al^[29]^ conducted a retrospective nationwide study in Danish individuals and disclosed that metformin combined with insulin had higher risk (rate ratios = 1.95) of all-cause mortality as compared with metformin plus SU. Our results were consistent with these 2 studies that early initiation of insulin after metformin failure, as compared with glimepiride, had high risk of all-cause mortality, CV and noncardiovascular death, after adjusting for all comorbidities and diabetes severity in a Chinese population.

The reasons why insulin might increase CV events and mortality in type 2 diabetes were many, including: insulin use might increase in body weight, raise the risk of hypoglycemia, and arrhythmias;^[30]^ exogenous insulin using would increase insulin resistance and hyperinsulinemia,^[31]^ which could exacerbate vascular inflammation,^[32]^ alter vascular hemodynamic,^[33]^ coagulopathy,^[34]^ and cellular mitogenicity.^[35]^

Our cohort also disclosed that male (aHR = 1.43) and old-aged diabetic persons (>65 year old, aHR = 2.31) had higher risk of all-cause mortality, which were consistent with Taiwan's nationwide survey.^[36]^ This cohort also showed that AGIs had lower risk of all-cause mortality (aHR = 0.48). Hanefeld conducted a meta-analysis of 7 long term studies and reported that acarbose could prevent myocardial infarction and CV disease in type 2 diabetic patients though most of them were already on intensive concomitant CV medication.^[37]^ Our study also revealed that DPP-4 inhibitors using had lower risk of mortality, which was consistent with Monami meta-analysis.^[38]^ But these 2 extra findings might need more rigorous matching study of insulin and other possible confounding factors to clarify them in the real word practice.

Our study had some strength. First, this was a population-based design and a real world finding, a 9-year follow-up data collected from the national insurance database. Second, the database contained a highly representative specimen of Taiwan's general population. Ninety nine percent of the entire 23 million people were enrolled in the national health insurance program. Third, we well matched the control group by using a propensity score calculated from age, gender, c*omorbidities*, other oral antidiabetic drugs, DCSI scores, and diabetes duration to reduce probable confounding.

Nevertheless, our study was subjected to a few limitations. First, the NHIRD did not give patients’ information about lifestyle, physical activity, smoking habits, and family history; all were possible confounding factors in this study. To avoid this bias, we matched the DCSI scores, duration of diabetes, comorbidities, and other oral antidiabetic drugs to abate the influence of disease severity. Second, the database was dearth of biochemical blood test results that could tell us the treated condition of patients. Finally, this study was an observational cohort study instead of a randomized controlled trial. The results required further prospective clinical trials to verify.

## 5 Conclusions

In summary, our study disclosed that insulin vs glimepiride in patients with background metformin therapy had higher risk of all-cause mortality. For the most of patients with type 2 diabetes, there was no unambiguous evidence of benefit from insulin. Early insulin treatment in type 2 diabetic patient might associate with unacceptable risks.^[39]^ However, insulin is the only option available to control blood glucose levels in the advanced stage of diabetes.

## Acknowledgments

This manuscript was edited by Wallace Academic Editing.

## Author contributions

**Conceptualization:** Fu-Shun Yen.

**Data curation:** Yuan-Chih Su, James Cheng-Chung Wei, Chii-Min Hwu.

**Writing – original draft:** Fu-Shun Yen, Chii-Min Hwu.

**Writing – review & editing:** Chih-Cheng Hsu, Yuan-Chih Su, James Cheng-Chung Wei.
